# Immune checkpoint inhibitor‐related molecular markers predict prognosis in extrahepatic cholangiocarcinoma

**DOI:** 10.1002/cam4.6441

**Published:** 2023-10-10

**Authors:** Bao Jin, Yuxin Wang, Baoluhe Zhang, Haifeng Xu, Xin Lu, Xinting Sang, Wenze Wang, Yilei Mao, Pengxiao Chen, Shun Wang, Zhirong Qian, Yingyi Wang, Shunda Du

**Affiliations:** ^1^ Department of Liver Surgery, Peking Union Medical College Hospital Chinese Academy of Medical Sciences& Peking Union Medical College Beijing China; ^2^ Department of Pathology, Peking Union Medical College Hospital Chinese Academy of Medical Sciences & Peking Union Medical College Beijing China; ^3^ Beidou Precision Medicine Institute Guangzhou China; ^4^ Department of Medical Oncology, Peking Union Medical College Hospital Chinese Academy of Medical Sciences & Peking Union Medical College Beijing China

**Keywords:** extrahepatic cholangiocarcinoma, microsatellite instability, overall survival, tumor mutational burden, whole‐exome sequencing

## Abstract

**Background:**

Therapeutic approaches for extrahepatic cholangiocarcinoma (EHCC) are limited, due to insufficient understanding to biomarkers related to prognosis and drug response. Here, we comprehensively assess the molecular characterization of EHCC with clinical implications.

**Methods:**

Whole‐exome sequencing (WES) on 37 tissue samples of EHCC were performed to evaluate genomic alterations, tumor mutational burden (TMB) and microsatellite instability (MSI).

**Results:**

Mutation of KRAS (16%) was significantly correlated to poor OS. ERBB2 mutation was associated with improved OS. ERBB2, KRAS, and ARID1A were three potentially actionable targets. TMB ≥10 mutations per megabase was detected in 13 (35.1%) cases. Six patients (16.2%) with MSIsensor scores ≥10 were found. In multivariate Cox analysis, patients with MSIsensor sore exceed a certain threshold (MSIsensor score ≥0.36, value approximately above the 20th percentile as thresholds) showed a significant association with the improved OS (HR = 0.16; 95% CI: 0.056–0.46, *p* < 0.001), as well as patients with both TMB ≥3.47 mutations per megabase (value approximately above the 20th percentile) and MSIsensor score ≥0.36.

**Conclusions:**

TMB and MSI are potential biomarkers associated with better prognosis for EHCC patients. Furthermore, our study highlights important genetic alteration and potential therapeutic targets in EHCC.

## 1 INTRODUCTION

As a fatal tumor of the bile ducts, extrahepatic cholangiocarcinoma (EHCC) has limited therapeutic options with an overall poor prognosis.^1^, ^2^ FDA has approved several targeted therapies for cholangiocarcinoma (CCA), including drugs that target *IDH1* mutation and fibroblast growth factor receptor 2 (*FGFR2*) fusions in genetically selected populations.^3^, ^4^ However, these FDA‐recognized gene alterations were not significant in EHCC, where *TP53* and *KRAS* genes were identified to have high‐frequency mutations.^5^, ^6^, ^7^ So far, few targeted therapies for EHCC have been approved, thus identification of possible therapeutic targets represents high unmet needs. However, the association between genetic alterations in EHCC and prognosis are not fully investigated.

Immune checkpoint inhibitors (ICIs) aimed at providing clinical benefit to CCA are currently under investigation. High TMB and MSI might be related to prolonged progression‐free survival (PFS) and overall survival (OS) in a variety type of tumors.^8^, ^9^, ^10^ Notably, various cut‐offs for the definition of TMB‐high and MSI‐high resulted in a highly varied proportion of TMB‐high and MSI‐high tumors in EHCC among different researches.^11^, ^12^ Moreover, the predictive role of TMB‐high and MSI‐high in discriminating the responders to ICIs among CCA patients remains elusive.^12^, ^13^, ^14^ Thus, the optimal TMB and MSI thresholds that associated with better survival are needed to be preferentially identified, which would favor to understand correlations of TMB and MSI with clinical outcomes of ICIs in EHCC.

To make a further comprehensive analysis of EHCC molecular profiling and understand the associations of mutation traits with clinical translational significance, we performed a comprehensive analysis of whole exome sequencing from a Chinese cohort of 37 patients with EHCC. We revealed the somatic mutation landscape of Chinese patients with EHCC, identified optimal TMB and MSI threshold associated with better survival, and explored novel actionable target and mutational signatures linked with the etiological background.

## 2 MATERIALS AND METHODS

### 2.1 Patients and tumor samples

This research was approved by the Ethics Committee of Peking Union Medical College Hospital (PUMCH). Briefly, patients who diagnosed with EHCC and underwent surgery without radiotherapy, chemotherapy or immunotherapy at PUMCH were enrolled from January 2013 to December 2017. Two experienced pathologists independently evaluated all histological specimens. Tumor samples and adjacent noncancerous tissue from 37 eligible patients with EHCC were collected in compliance with informed consent.

### 2.2 Clinical data

Information about age, sex, tumor anatomic location, histological subtype of tumor, tumor stage, and surgical margins were obtained. Overall survival was defined as time from surgery until death from any causes. The ultimate vital states of all 37 patients were death during follow‐up.

### 2.3 Whole‐exome sequencing

Using the GeneRead DNA FFPE Kit, genomic DNA was extracted from formalin‐fixed paraffin‐embedded (FFPE) samples and then was broke into ~250 bp fragments through M220 Focused‐ultrasonicator (Covaris). SureSelect Human All Exon V6 Kit (Agilent Technologies) was used for whole genome library preparation and exome capture.^15^ Prepared libraries were sequenced on Illumina HiSeq 6000 platform. Mean coverage depth of ~80× for the normal control (adjacent noncancerous tissue) and ~250× for the tumor samples were attained.

### 2.4 Analysis of SNV and INDEL calling

High‐quality clean data were obtained by discarding reads with adapter contamination, low‐quality nucleotides and more than 10% uncertain nucleotides. Paired‐end clean reads were mapped to the reference genome (UCSC hg19) with Burrows–Wheeler Aligner (BWA) software (https://bio‐bwa.sourceforge.net/, RRID:SCR_010910).^16^ Duplicates originated from PCR amplification was marked via Genome Analysis Toolkit (https://software.broadinstitute.org/gatk/, RRID:SCR_001876).^17^ According to instructions of GATK best practice,^18^ Indelrealigner and RealignerTargetCreator in GATK toolkits were utilized to perform reads realignment around regions of apparent indels. Base quality score recalibration (BQSR) process was then conducted using GATK BaseRecalibrator and ApplyBQSR. Short somatic variants (SNPs and INDELs) were identified and filtered using Strelka2 between tumor and matched adjacent noncancerous samples. Detected mutations were then annotated with ANNOVAR (http://www.openbioinformatics.org/annovar/, RRID:SCR_012821). All annotated variants were gathered and summarized using the R package maftools.

### 2.5 Copy number variation detection

Copy number variations were detected using CNV‐Facets,^19^ and tumor purity and ploidy levels were estimated simultaneously. CNV hotspots were detected, and CNV results were summarized using GISTIC2(http://www.mmnt.net/db/0/0/ftp‐genome.wi.mit.edu/distribution/GISTIC2.0,RRID:SCR_000151).^20^

### 2.6 Mutation spectrum and mutation signature analysis

To extract single base substitutions (SBS) signatures, a matrix of mutational spectrum with 96 elements (based on six base substitutions (C>A, C>G, C>T, T>A, T>C, and T>G) within 16 possible combinations of up and down stream bases for each substitution) was extracted from somatic SNVs using Mutational Signatures in Cancer (MuSiCa) software. Then *de*‐*novo* SBS signatures were extracted using nonnegative matrix factorization (NMF) method in MuSiCa.^21^ Cosine similarity between de‐novo SBS signatures and 30 known COSMIC cancer signatures (https://cancer.sanger.ac.uk/cosmic/signatures) were calculated to illustrate potential functions and ontologies of these signatures. For each sample, proportions of signatures were calculated using MuSiCa and samples were then clustered.

### 2.7 TMB and MSI analysis

For each sample, TMB was uniformly calculated as the number of nonsynonymous mutations per megabase (Mb) of targeted exomic regions with at least 50× coverage.

To determine the MSI status, MSIsensor algorithm (https://github.com/ding‐lab/msisensor, RRID:SCR_006418) was applied^22^ to calculate the percentage of unstable microsatellite loci in the tumor genome compared to its matched normal genome. The reference genome was systematically scanned by MSIsensor to identify and mark microsatellite sites. Subsequently, the distribution of simple repeat elements covering these microsatellites was calculated in both the normal and tumor samples. Microsatellite instability was inferred by assessing the differences in the distribution of repeat elements using the Chi‐Squared test. The proportion of unstable microsatellites was then computed, and the MSI level was determined by examining the distribution of unstable microsatellite proportions across all cohorts. MSIsensor score ≥10 was validated in colorectal cancers to separate MSI‐H from MSS tumors identified by IHC and/or PCR with a high accuracy.^23^

### 2.8 Statistical analysis

Median OS was analyzed by the Kaplan–Meier method and compared using the log‐rank test. The association of mutation with OS was analyzed using univariate analysis and multivariate analysis with the Cox proportional hazards model. Age, gender, pathologic stage, differentiation, lymphatic metastasis, margin, habit of drinking alcohol and smoking were added as covariates in univariate and multivariate Cox analysis. Among the above variates, *p* value of age and differentiation was <0.05. Resection margins status and tumor stage are previously reported prognostic factors affecting survival of EHCC.^24^ To make sure variables were weighed, age, pathologic stage, differentiation, and margin were selected as variates for analysis of cancer related genes, TMB and MSI associated with EHCC overall survival using multivariate Cox proportional hazards regression, even though the *p* values of margins status and tumor stage were not significant. For continuous variables, hypothesis testing was performed by Student's paired *t*‐tests or Wilcoxon matched‐pairs signed‐rank test. Statistical analyses were carried out using R (v.3.4.1). A 2‐side *p* value <0.05 was considered statistically significant.

## 3 RESULTS

### 3.1 Clinicopathological characteristics and sequence data of patients with EHCC

The cohort contained tumor samples of 37 therapy naïve cases from PUMCH (Table S1). The demographics and clinicopathological information are shown in Table 1. The longest survival time of patients in this cohort was 50 months, the shortest was 4 months, and the median was 23 months. Age, disease stage, tumor differentiation, and margins were correlated to OS in univariate Cox regression analysis (*p* < 0.2). Thus, these factors were selected as covariates for adjusting the subsequent gene alterations in the multivariate Cox proportional hazards regression model. As anticipated, poorly tumor differentiation was associated with shorter OS (Table 1).

**TABLE 1:** Baseline patient and tumor characteristics (*n* = 37).

Characteristic	No. of patients	%	Univariate HR	95% CI	*p*	Multivariate HR*	95% CI	*p*
Age	37		1.1	1–1.1	0.0081	1.05	1.0–1.1	0.065
Mean	60 ± 8.83							
Median	61							
Gender	37							
Female	21	56.8	1	Reference	–	1	Reference	–
Male	16	43.2	0.93	0.48–1.8	0.84	0.68	0.25–1.8	0.441
Stage at diagnosis								
I	9	24.3	1	Reference	–	1	Reference	–
II	22	59.4	1.1	0.51–2.4	0.82			
III	6	16.2	4.2	1.5–11	0.0051	3.66	0.75–17.8	0.107
Differentiation								
High	8	21.6	1	Reference	–	1	Reference	–
High or moderate	7	18.9	1.9	0.85–4.2	0.12			
Moderate	8	21.6	3.2	1.6–6.6	0.0013	4.34	1.69–11.2	0.002
Moderate or low	10	27.0	4.3	2–9.3	0.00025			
Low	4	10.8	1.9	0.66–5.5	0.24			
Lymphatic_Metastasis								
No	26	70.3	1	Reference	–	1	Reference	–
Yes	11	29.7	1.3	0.63–2.7	0.48	0.81	0.25–2.6	0.729
Margin								
Negative	25	67.6	1	Reference	–	1	Reference	–
Positive	12	32.4	1.9	0.94–3.9	0.076	0.89	0.34–2.3	0.807
Alcohol								
Never	7	18.9	1	Reference	–	1	Reference	–
Ever	30	81.1	0.64	0.27–1.5	0.3	0.70	0.20–2.4	0.568
Smoking								
Never	14	37.8	1	Reference	–	1	Reference	–
Ever	23	62.2	1	0.53–2	0.9	2.60	1.0–6.8	0.051

Abbreviation: HR, hazard ratio.

* The multivariate Cox proportional hazards regression model was adjusted for age, gender, pathologic stage, differentiation, Lymphatic Metastasis, Margin, habit of drinking alcohol, and smoking.

### 3.2 The landscape of mutation in Chinese patients with EHCC

**FIGURE 1:**
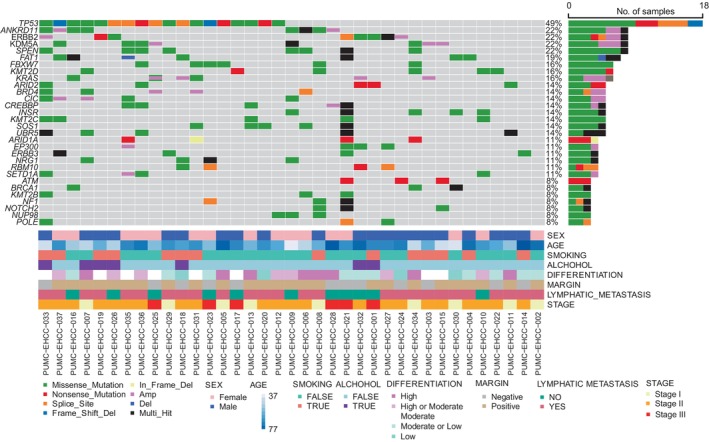
The landscape of somatic alterations in cancer related genes annotated by OncoKB. The cancer‐related genes annotated by OncoKB with the mutations are listed on the left‐side and the mutation status of the recurrently mutated genes for each tumor was showed in middle plot. Each column represents a sample. The right bar plot summarizes the ratio of different mutation types. Different colors refer to mutational types and clinicopathologic characteristics at low panel.

In total, 8962 somatic mutations and 7517 nonsynonymous somatic mutations in 5627 genes were identified from the 37 patients with EHCC by whole‐exome sequencing (Figure S1; Table S2). The median number of mutations per sample was 112. The most frequently altered gene was *MUC16* (49%), followed by *TP53* (49%), *TTN* (43%), *MUC4* (30%), and *OBSCN* (24%) (Figure S1; Table S2). The genes with a mutation frequency greater than 16% contained six tumor suppressor genes and three oncogenic genes annotated by OncoKB (www.oncokb.org),^25^ including *TP53* (48.6%), *ANKRD11* (22%), *ERBB2* (22%), *SPEN* (22%), *KDM5A* (22%), *FAT1* (19%), *FBXW7* (16%), *KMT2D* (16%), and *KRAS* (16%), among which CNV‐driving alterations were observed in the oncogenic genes *ERBB2*, *KDM5A*, and *KRAS* and tumor suppressor genes *ANKRD11* and *FAT1* (Figure 1). The alterations of KRAS included mutation and amplification, with a frequency of 16.2% in our cohort (Table 2). After adjusting for age, disease stage, tumor differentiation, and margins in a Cox proportional hazards model, mutations in *ERBB2* were significantly associated with better prognosis (a median of 27 months with *ERBB2* mutant tumor, 95% CI: 4.8–49.2 months vs. a median of 20 months with *ERBB2* wild‐type tumor, 95% CI: 14.7–25.3 months), and mutations in *KRAS* were significantly associated with worse prognosis (a median of 15 months with *KRAS* mutant tumor, 95% CI: 9.4–20.6 months vs. a median of 27 months with *KRAS* wild type tumor, 95% CI: 19.5–34.5 months), which showed in Table 2; Table S3.

**TABLE 2:** Analysis of cancer‐related genes, TMB and MSI associated with EHCC overall survival.

Variable		No. of patients	%	Multivariate HR* (95% CI)	*p*
ERBB2	WT	29	78.4	Reference	
	Mutant & amplification	8	21.6	0.2 (0.077–0.54)	0.001^##^
KARS	WT	31	83.8	Reference	
	Mutant & amplification	6	16.2	3.6 (1.29–10.2)	0.014^#^
TMB	Bottom 20%	8	21.6	Reference	
	Top 80%	29	78.4	0.46 (0.16–1.3)	0.14
MSI	Bottom 20%	8	21.6	Reference	
	Top 80%	29	78.4	0.16 (0.056–0.46)	<0.001^##^
MSI_TMB	Other	13	35.1	Reference	
	MSI top 80% & TMB Nonsynonymous 80%	24	64.9	0.21 (0.079–0.58)	0.002^##^

Abbreviation: HR, hazard ratio.

* The multivariate Cox proportional hazards regression model was adjusted for covariates with *p* < 0.05 in univariate analysis and variables that are known to affect prognosis of EHCC even though the *p* values were not significant here, which including age, pathologic stage, differentiation, and margin.

^#^ *p* < 0.05 in multivariate Cox proprotional hazards analysis.

^##^ *p* < 0.01 in mutivariate Cox proportional hazards analysis.

### 3.3 Actionable genomic alterations and targeted therapies

A total of 29.7% of patients (11/37) had one actionable genetic alteration that mainly caused gain of function and truncating mutations, which were classified as level 3B or 4 according to OncoKB classification (Table 3). These included oncogenic somatic alterations in *ERBB2* (three patients), *KRAS* (three patients), *ARID1A* (three patients), *PIK3CA* (one patient), and *SF3B1* (one patient). *ERBB2* amplification was also observed in two patients (Figure 1; Table 3).

**TABLE 3:** Potential actionable alteration levels of 37 EHCC sample in OncoKB database.

Actionable gene	Alteration	Number of patients	Mutation effect	Therapeutic level	Drug
ERBB2	S310F	2	Gain‐of function	3B	Ado‐Trastuzumab Emtansine Trastuzumab Deruxtecan Neratinib
	R678Q	1	Gain‐of‐function		
ARID1A	R1989X	3	Truncating Mutations	4	Tazemetostat PLX2853
KRAS	G12V	1	Gain‐of‐function	3B/4	Adagrasib Cobimetinib Binimetinib Trametinib
	G13D	1	Gain‐of‐function		
	A146V	1	Gain‐of‐function		
PIK3CA	E545K	1	Gain‐of‐function	3B	Alpelisib + Fulvestrant
SF3B1	K700E	1	Switch‐of‐function	4	H3B‐8800

### 3.4 TMB and MSI are associated with improved OS

The association between nonsynonymous somatic TMB and OS of EHCC was examined. Because the median and range of TMB vary across tumor types, a given numeric value of TMB, such as patients whose TMB ≥10, which is grouped as high TMB in NSCLC cohorts, is not applicable as a universal cut‐off for other type of tumor.^26^ In our cohort, 35.1% of patients (*n* = 13) have tumors with TMB ≥10 (Table S1). We stratified tumors by TMB decile and identified TMB value thresholds by investigating the association between TMB subgroups and the OS of patients under univariate Cox analysis. We found that the cut‐off value of TMB in the range among the top 90%, 80%, and 70% could effectively separate patients with high and low level of TMB (Table S4). In addition, a significant association was found between the OS of EHCC and a variety of cut‐off points defined by the decile of the MSIsensor score (Table S5). The top 80% of TMB values (≥3.47 mutations per megabase) and top 80% of MSIsensor score (MSIsensor score ≥0.36), both of which showed the lowest *p* values in univariate Cox analysis, were served as a watershed to separately divide the patients into two subgroups for the subsequent multivariate analysis. Typically, MSIsensor score ≥ 10 was defined as MSI‐high which showed a high concordance in validation by MSI PCR and/or MMR immunohistochemistry for colorectal cancers and uterine endometrioid cancer.^23^ In our cohort, six patients (16.2%) with MSIsensor scores ≥10 were found.

**FIGURE 2:**
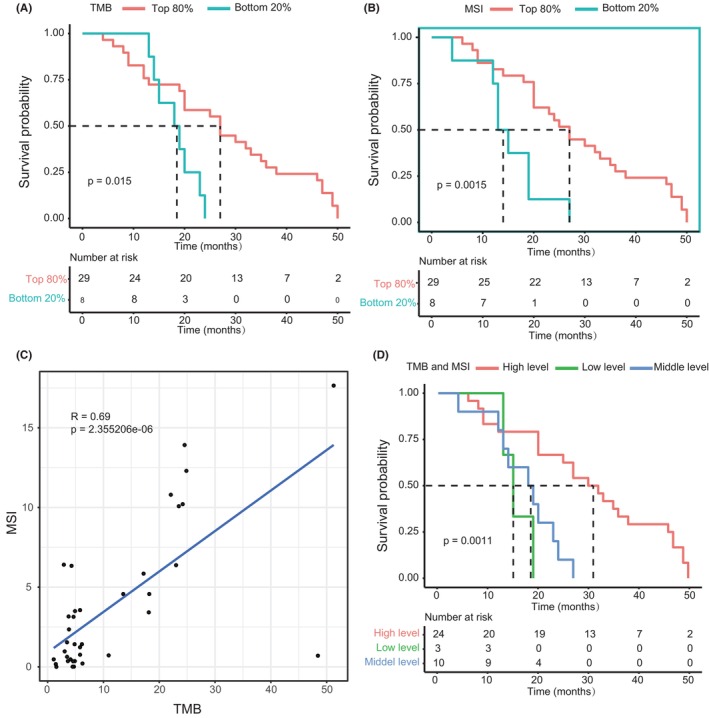
Improved OS in patients with high TMB and high MSI. (A) OS curves of top 80% of TMB and bottom 20% of TMB groups (*p* = 0.015). (B) OS curves of top 80% of MSIsensor score and bottom 20% of MSIsensor score groups (*p* = 0.0015). (C) The positive relation of MSI and TMB (*R* = 0.69). (D) OS curves of three TMB and MSI status (*p* = 0.001). Log‐rank test was used to calculate *p* value. OS, overall survival.

The mean OS of TMB ≥3.47 versus TMB <3.47 was 27.6 months (95% CI: 22.1–33.1) versus 18.2 months (95% CI: 15.4–21.1), respectively, and the median OS of TMB ≥3.47 versus TMB <3.47 was 27 months (95% CI: 23.5–30.5) versus 18 months (95% CI: 12.5–23.5), respectively. It was found that TMB ≥3.47 was significantly associated with improved OS (log‐rank test, *p* = 0.015) (Figure 2A). However, in multivariate Cox analysis, TMB ≥3.47 did not show a significant association with the OS of EHCC (Table 2, HR = 0.46, 95% CI: 0.16–1.3, *p* = 0.14).

The mean OS of top 80% of MSIsensor score versus bottom 20% of MSIsensor score was 28.4 months (95% CI: 23.3–33.6) versus 15.3 months (95% CI: 10.6–19.9), respectively. The OS of EHCC was significantly longer in patients with top 80% of MSIsensor score (median of 27 months, 95% CI: 21.7–32.2) than in those with bottom 20% of MSIsensor score (median of 13 months, 95% CI: 10.2–15.8, Figure 2B, log‐rank test, *p* = 0.0015). We observed a 6.25‐fold decrease in mortality risk for cases with top 80% of MSIsensor score in multivariate Cox proportional hazards model (HR, 0.16; 95% CI: 0.056–0.46; *p* < 0.001; Table 2).

Nevertheless, when both TMB and MSI were enrolled as confounders in the multivariate analysis of OS (Table S6), top 80% of MSIsensor score was significantly correlated to OS, and top 80% of TMB values was not, indicating that TMB was not significantly associated with OS at a circumstance of MSI as a covariate. Then, we checked the collinearity of these two factors. A considerable collinearity relationship was observed between TMB and MSI (*R* = 0.69, *p* = 2.355206e‐06, Figure 2C). In line with that, both TMB and MSI belong to the top 80%, named as MSI_TMB, was significantly associated with lower mortality risk, as determined by multivariate Cox analysis (HR, 0.21; 95% CI: 0.079–0.58; *p* < 0.002; Table 2). To further dissect the effect of TMB and MSI on OS, we divided patients into three different groups as follows: (1) high level: both TMB and MSI belong to the top 80%; (2) low level: both TMB and MSI belong to the bottom 20%; and (3) middle level: others. The difference in OS was found significant among these groups (log‐rank test, *p* = 0.001, Figure 2D), showing that patients with both TMB and MSI belong to the top 80% had the best OS.

### 3.5 Mutational signatures of Chinese patients with EHCC

**FIGURE 3:**
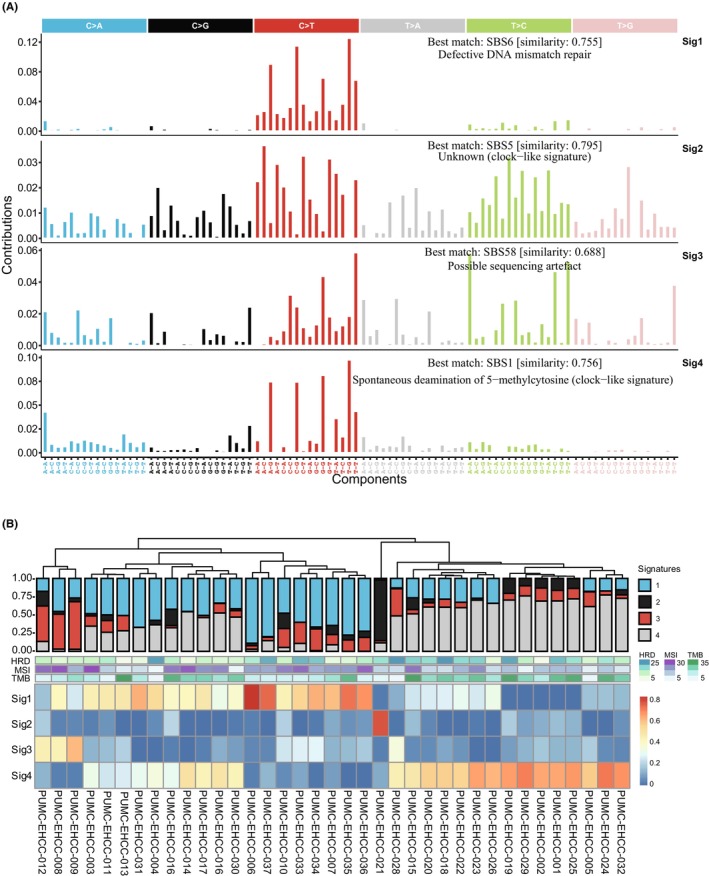
Profile of mutational signatures in 37 EHCC patients. (A) Four mutation signatures detected in EHCC (sig1–sig4), annotated with the corresponding COSMIC signature determined by cosine similarity. Sig 1 (COSMIC signature 6, cosine similarity = 0.755), Sig2 (COSMIC signature 5, cosine similarity = 0.792), Sig3 (COSMIC signature 58, cosine similarity = 0.686), and Sig4 (COSMIC signature 1, cosine similarity = 0.753). (B) Proportions of somatic mutations in 4 mutation signatures for each individual (top) and status of HRD, MSI and TMB enrichment (middle). The samples clustered by hierarchical cluster analysis according to the proportional contribution of each signature per sample (bottom). Sig, signature.

Further, four mutational signatures were identified (Figure 3A). By comparing with the COSMIC signatures, we found that signature 1 is similar to COSMIC signature S6, which is related to defective DNA mismatch repair (similarity = 0.755). Signature 4 is similar to COSMIC signature S1, which is related to spontaneous deamination of 5‐methylcytosine (similarity = 0.753). Signatures 2 and 3 were similar to COSMIC signatures S5 and S58, respectively, which are related to unknown mutation processes or sequencing artifacts. The proportions of somatic mutations in the four mutation signatures for each individual and the corresponding TMB and MSI values are shown in Figure 3B. The samples clustered according to the proportional contribution of each signature per sample and were divided into two signature clusters, with signature 1 and signature 4 dominating each (Figure 3B).

COSMIC signature 6 is strongly associated with the inactivation of DNA mismatch repair genes in colorectal cancer. Furthermore, we studied the clinical implication of DDR‐related genes in EHCC. In particular, we focused on the DNA damage repair pathway, and the DDR gene list was from the work of Arai et al.^27^ A total of 40.5% of patients (15/37) had 19 nonsynonymous mutations in DDR‐related genes, among which *ATM* (8%), *CDK12* (8%), and *BRCA1* (8%) were the most frequently mutated genes. All the alterations in *ATM* were nonsense mutations (Figure S2A), and *ATM* mutations showed a significant association with better OS (Table S7). There was no significant difference between HRD scores of the DDR mutant and wild‐type (*t*‐test, *p* = 0.92). The association of the mutation in a group of DNA damage repair genes with survival probability was analyzed by multiple value Cox analysis (Figure S2B). The results showed that mutations in a group of DNA damage repair genes did not significantly influence the survival probability of EHCC (HR = 0.59, 95% CI: 0.14–2.4, *p* = 0.462).

## 4 DISCUSSION

The incidence rate of EHCC in the Asian population is higher than that in Europe and America, and its pathogenesis is not clear.^28^ It is also a challenge to predict and improve the clinical outcome of patients with EHCC, possibly due to sample rarity, tumor heterogeneity, poorly understood pathophysiology, and lack of actionable driver events.^29^ Here, we described the genomic characteristics of Chinese patients with EHCC and unraveled distinct genomic features with potential clinical implications, optimal TMB and MSI threshold associated with better survival, mutational signatures related to etiology and potentially actionable targets. These results enlighten precise treatment strategies for EHCC.

In this study, we reported for the first time that 49% of patients had *MUC16* mutations. *MUC16* is an important biomarker for the early diagnosis of epithelial ovarian cancer.^30^ The abnormal expression of *MUC16* is remarkably associated with the poor prognosis of many cancers.^31^, ^32^ Recently, it is reported that patients with *MUC16* mutations have a higher tumor mutation load and neoantigen load, and are more responsive to immunotherapy.^33^, ^34^ Besides, the combined mutation status of *MUC4*, *MUC16*, and *TTN* showed the potential to predict TMB and immunotherapy efficacy in gastric cancer and pan cancer.^35^ The alteration frequencies of *TTN* (43%) and *MUC4* (30%) in EHCC were also considerably notable in our study, suggesting that *MUC4*, *MUC16*, and *TTN* may be associated with immunotherapy efficacy of EHCC.

Many previous studies have proven that *TP53*, *KRAS*, *ERBB2*, and *SMAD4* have high mutation frequencies in EHCC,^5^, ^6^, ^7^, ^36^ among which alterations in *TP53* and *KRAS* have been reported to be significantly associated with poor prognosis.^36^, ^37^, ^38^, ^39^ Similarly, in our cohort, the mutation frequencies of *TP53* (49%), *ERBB2* (22%), and *KRAS* (16%) were relatively high compared with those of other mutated genes. Although the mutation frequency of *KRAS* in our cohort was lower than that in other researches,^5^, ^6^, ^7^, ^40^ alteration of *KRAS* was significantly associated with the poor prognosis of EHCC. Moreover, mutation of ERBB2 in our study was positively related to a longer OS of EHCC, which is contrary to its oncogenic mutation type found in our cohort.

To date, it is still lack of targeted agents for patients with EHCC. We found *ERBB2* and *KRAS* mutation in EHCC were actionable targets. *ERBB2* and *KRAS* are key genes in the RTK‐RAS pathway, and both of them could be classified as level 3B actionable genomic alterations in OncoKB, defined as standard care or investigational biomarkers predictive of response to an FDA‐approved or investigational drug in another indication.^41^ Level 1 genetic alterations predictive of response to an FDA‐approved drug in CCA are oncogenic mutations in *IDH1*, *FGFR2* fusions, and *NTRK1* fusions. However, these genetic alterations were not found in our study.

The main hotspot mutation of *ERBB2* in our cohort was the S310F missense mutation, which is an active mutation sensitive to irreversible dual EGFR/HER2 inhibitors.^42^ Anti‐HER2 targeted therapy in patients with ERBB2 amplification have shown effectiveness in cholangiocarcinoma.^43^ At present, a clinical trial (NCT03093870) is ongoing to investigate the safety and efficacy of capecitabine in combination with varlitinib, an inhibitor of EGFR, HER2 and HER4, for the treatment of biliary tract cancer. Molecular profiling for EHCC highlighted that EHCC is enriched with actionable mutations. Genomic‐driven targeted therapy is promising for improving patient outcomes and contributing to the clinical management of patients suffering with refractory EHCC.

Unexpectedly, we also demonstrated that the top 80% of MSIsensor score (MSIsensor score ≥0.36) in our cohort can be used as a prognostic indicator for patients with EHCC. MSIsensor score of 35.1 was found in one tumor (0.5%) and identified as MSI‐high from a cohort of 195 CCA patients (78% intrahepatic and 22% extrahepatic cholangiocarcinoma).^44^ Recently, the percentages of PD‐L1 overexpression, MSI‐High, and TMB‐High were investigated in CCA, in which EHCCs had a lower prevalence (6.9%).^11^ TMB and MSI levels have been shown to be associated with the immunotherapy efficacy and prognosis of various cancers. Although the prognostic significance of MSI‐High has been reported in CCA,^45^ whether TMB and MSI contribute to the survival of EHCC has not been reported. The median TMB in our cohort was 4.91 Mut/Mb, which was higher than the previously reported median TMB determined by WES, which was 1.23 (0.7–2.34) Mut/Mb in CCA.^39^ 29.4% of patients have tumors with TMB ≥10 muts/Mb are grouped as TMB‐High in NSCLC cohorts.^26^ In a study of 3634 CCA patients, only 118 and 47 cases had TMB >10 and >20 mut/mb, respectively.^46^ Besides, MSI‐high was rare in CCA (1%) and EHCC (0%–2%).^6^, ^11^, ^46^ In our cohort, a TMB ≥10 muts/Mb was seen in 35.1% of tumors (*n* = 13). The percentage of TMB ≥10 mut/mb in our research was higher than previous research results of EHCC (7.5%),^6^ which may attribute to small sample size of our study. After multivariate analysis, we found that the top 80% of MSIsensor score was significantly correlated with prognosis, whereas there was no significant association between top 80% of TMB in our cohort and prognosis. Our study showed that patients with both TMB and MSI belong to the top 80% had longer OS. Therefore, it needs to be further demonstrated whether TMB can be used to judge the prognosis of patients with EHCC, and it is valuable to study whether EHCC patients with TMB‐High and MSI‐High are responsive to immune checkpoint inhibitors targeting PD‐1/PD‐L1.

In cancer genome, different mutational signatures formed during a variety of mutational processes, which were strongly associated with the epidemiological and biological features of particular cancer types. By comparison in COSMIC databases, the major mutation signature in our cohort is COSMIC signature 6, which is associated with DNA mismatch repair defects.^47^ MSI can result from defective DNA mismatch repair, and MSI‐high was strongly associated with prolonged survival in our cohort. However, the mutated DNA damage repair genes in our study were not prognostic. Along with the previous observation in cholangiocarcinoma,^39^, ^48^ we found that signature 4 is similar to COSMIC signature 1, which is related to the spontaneous deamination of 5‐methylcytosine.^47^ The study of these mutation signatures will be helpful to deepen the understanding of the etiology of EHCC in Chinese individuals.

In conclusion, our study found mutations in ERBB2 and KRAS were prognostic factors for the overall survival of EHCC. Besides, overall survival was significantly longer in patients with both TMB and MSI belong to the top 80%. We posed that ERBB2 was one of the most prospective actionable targets of EHCC. In our study, we present a broad perspective to understand the molecular traits of EHCC for Chinese patients and offer valuable information for further in‐depth study on EHCC. Due to a small sample size in our research might cause bias in the data, study of large EHCC cohort need to be conducted in future.

## AUTHOR CONTRIBUTIONS

**Bao Jin:** Data curation (equal); formal analysis (equal); investigation (equal); writing – original draft (equal). **Yuxin Wang:** Data curation (equal); formal analysis (equal); investigation (equal); writing – original draft (equal). **Baoluhe Zhang:** Data curation (equal); formal analysis (equal); investigation (equal). **Haifeng Xu:** Resources (equal). **Xin Lu:** Resources (equal). **Xin‐Ting Sang:** Resources (equal). **Wenze Wang:** Investigation (equal). **Yilei Mao:** Resources (equal). **Pengxiao Chen:** Validation (equal); visualization (equal); writing – review and editing (equal). **Shun Wang:** Validation (equal); visualization (equal); writing – review and editing (equal). **Zhirong Qian:** Conceptualization (equal); supervision (equal); validation (equal); visualization (equal). **Yingyi Wang:** Conceptualization (equal); funding acquisition (equal); resources (equal); supervision (equal). **Shunda Du:** Conceptualization (equal); funding acquisition (equal); resources (equal); supervision (equal).

## FUNDING INFORMATION

This work was supported by the National Natural Science Foundation of China (Grant No. 81972698) and Chen Xiao‐Ping Foundation for the Development of Science and Technology of Hubei Province (Grant No. CXPJJH11900001‐2019215).

## CONFLICT OF INTEREST STATEMENT

The authors have no conflict of interest to declare.

## ETHICS STATEMENT

The studies involving human participants were reviewed and approved by Medical Ethics Committee of Peking Union Medical College Hospital (PUMCH). Written informed consent was obtained from the individual(s), and minor(s)’ legal guardian/next of kin, for the publication of any potentially identifiable images or data included in this article.

## Supporting information


figure legend.

Table S1.

Table S2.

Table S3.

Table S4.

Table S5.

Table S6.

Table S7.

Figure S1.

Figure S2.

## Data Availability

Data sharing is not applicable to this article as no new data were created or analyzed in this study.
