# Supplementary material for: Immune checkpoint inhibitor‐related molecular markers predict prognosis in extrahepatic cholangiocarcinoma
Source: Cancer Med. 2023 Oct 10;12(20):20470–81. doi: 10.1002/cam4.6441 (PMC10652350; doi:10.1002/cam4.6441)
Supplement: Supplementary file 1 — figure legend. [file CAM4-12-20470-s006.docx]

**Supplementary Fig. 1** **The landscape of somatic alterations**

The genes with the mutations are listed on the left-side and the mutation status of the recurrently mutated genes for each tumour was showed in middle plot. Top bar plot summarizes the number of mutations per sample shown with corresponding clinicopathologic characteristics. Different colours refer to mutational types and clinicopathologic characteristics at low panel.

**Supplementary Fig. 2 Mutations in the DDR-related genes and impact of a DDR-related gene set on OS**

(A) Mutation status of DDR-related genes in EHCC was showed in middle plot. The ratio of mutation type in each gene was correspondently showed on the right panel. (B) OS curves of samples with or without mutated genes included in a DDR-related gene set. The contained genes of DDR-related gene set were presented on the plot.
