# Supplementary figures and images for: Immune checkpoint inhibitor‐related molecular markers predict prognosis in extrahepatic cholangiocarcinoma
Source: Cancer Med. 2023 Oct 10;12(20):20470–81. doi: 10.1002/cam4.6441 (PMC10652350; doi:10.1002/cam4.6441)

Altered in 37 (100%) of 37 samples

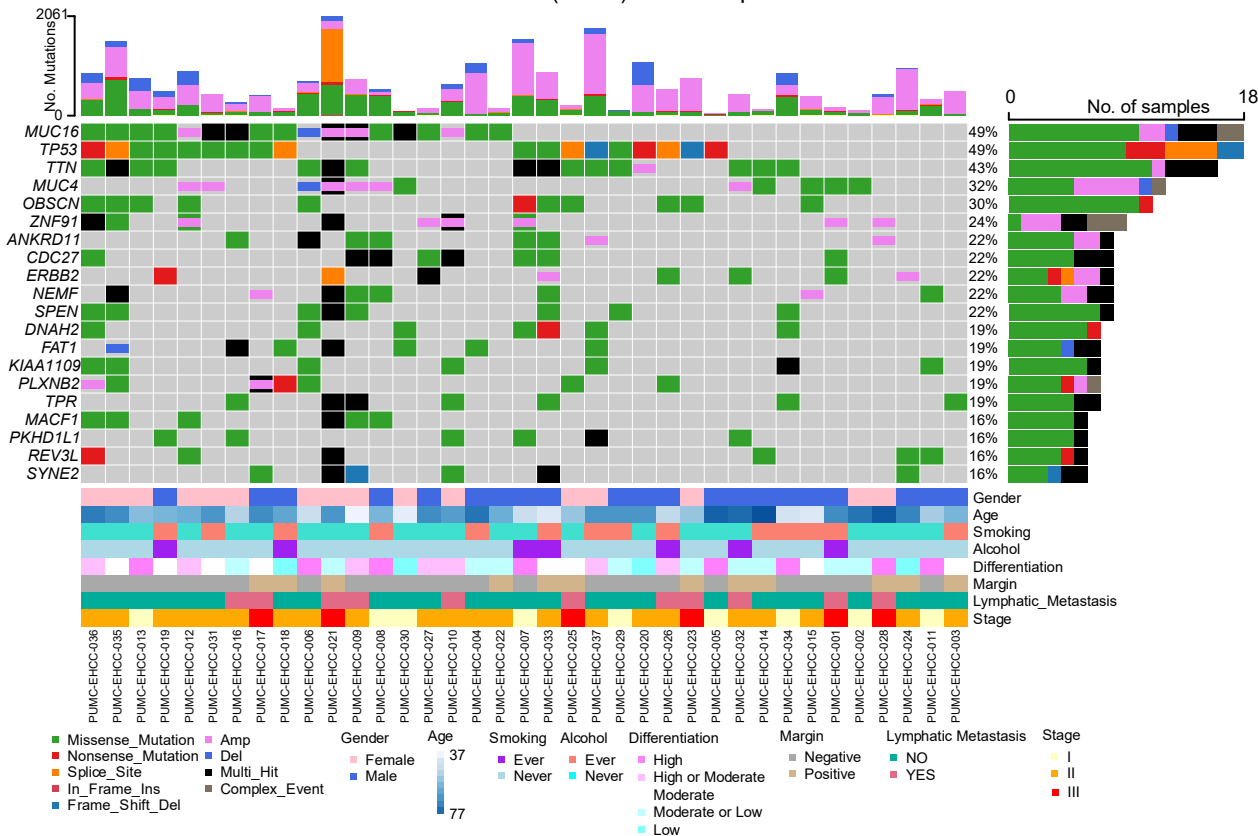

Supplement: Supplementary file 9 — Figure S1. [file CAM4-12-20470-s004.pdf]
