# Supplementary material for: Immune checkpoint inhibitor‐related molecular markers predict prognosis in extrahepatic cholangiocarcinoma
Source: Cancer Med. 2023 Oct 10;12(20):20470–81. doi: 10.1002/cam4.6441 (PMC10652350; doi:10.1002/cam4.6441)
Supplement: Supplementary file 10 — Figure S2. [file CAM4-12-20470-s001.pdf]

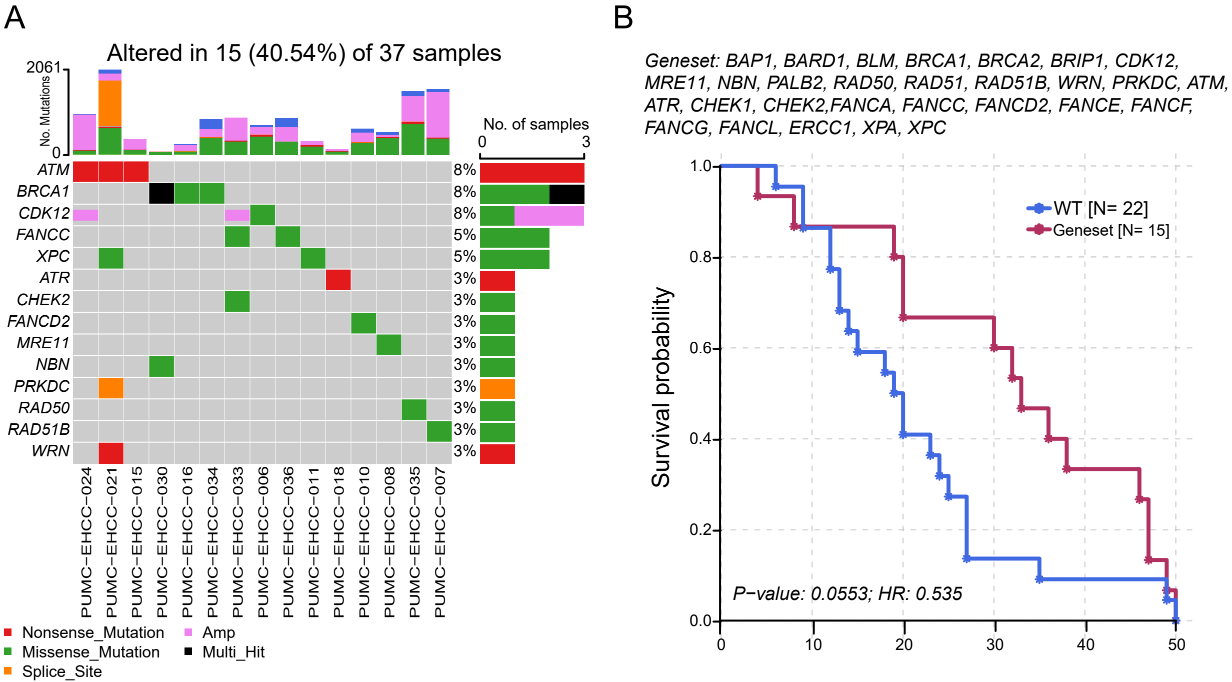
A
Altered in 15 (40.54%) of 37 samples
2061
No. Mutations
0
No. of samples
0
3
ATM
BRCA1
CDK12
FANCC
XPC
ATR
CHEK2
FANCD2
MRE11
NBN
PRKDC
RAD50
RAD51B
WRN
8%
8%
8%
5%
5%
3%
3%
3%
3%
3%
3%
3%
3%
3%
PUMC−EHCC−024
PUMC−EHCC−021
PUMC−EHCC−015
PUMC−EHCC−030
PUMC−EHCC−016
PUMC−EHCC−034
PUMC−EHCC−033
PUMC−EHCC−006
PUMC−EHCC−036
PUMC−EHCC−011
PUMC−EHCC−018
PUMC−EHCC−010
PUMC−EHCC−008
PUMC−EHCC−035
PUMC−EHCC−007
Nonsense_Mutation
Missense_Mutation
Splice_Site
Amp
Multi_Hit
B
Geneset: BAP1, BARD1, BLM, BRCA1, BRCA2, BRIP1, CDK12, MRE11, NBN, PALB2, RAD50, RAD51, RAD51B, WRN, PRKDC, ATM, ATR, CHEK1, CHEK2,FANCA, FANCC, FANCD2, FANCE, FANCF, FANCG, FANCL, ERCC1, XPA, XPC
WT [N= 22]
Geneset [N= 15]
Survival probability
1.0
0.8
0.6
0.4
0.2
0.0
0
10
20
30
40
50
P−value: 0.0553; HR: 0.535
